# Microarray dataset on the genome-wide expression profile of an *M. smegmatis amtR* mutant (JR258) compared to *M. smegmatis* mc^2^155

**DOI:** 10.1016/j.dib.2016.11.049

**Published:** 2016-11-23

**Authors:** Michael Petridis, Gregory M. Cook

**Affiliations:** a Department of Microbiology and Immunology, Otago School of Medical Sciences, University of Otago, Dunedin 9054, New Zealand; b Maurice Wilkins Centre for Molecular Biodiscovery, The University of Auckland, Private Bag 92019, Auckland 1042, New Zealand

## Abstract

The dataset presented here describes a microarray experiment to identify the AmtR regulon of *Mycobacterium smegmatis* comparing the transcription profile of a *M. smegmatis amtR* mutant to *M. smegmatis* wild-type. The raw and processed microarray data are available in the ArrayExpress database under Accession Number E-MTAB-4857 and interpretation of this data is found in the research article “Structure and function of AmtR in *Mycobacterium smegmatis*: implications for post-transcriptional regulation of urea metabolism through a small antisense RNA” (Petridis et al., in press) [1].

**Specifications Table**

**Table:** 

Subject area	*Biology*
More specific subject area	*Molecular biology*
Type of data	*Text file,.xlsx file (Excel)*
How data was acquired	*Scanned Genepix 4000A scanner. Images were quantified using Spotfinder (TIGR). Total normalization and LOWESS normalization with MIDAS software (TIGR)*
Data format	*Raw, processed*
Experimental factors	*A markerless deletion of the amtR gene in the background of M. smegmatis mc*^*2*^*155 was created.*
Experimental features	*Expression profiles of a M. smegmatis amtR mutant and M. smegmatis mc*^*2*^*155 were determined during aerobic growth in Hartmans de Bont minimal medium.*
Data source location	*Not applicable*
Data accessibility	*Data within this article are available in the ArrayExpress database (*https://www.ebi.ac.uk/arrayexpress/*) under Accession Number*E-MTAB-4857.

**Value of the data**

• Description of the AmtR regulon gives insight into its role in the regulation of nitrogen metabolism in actinomycetes.
• Our data can be used to further clarify gene regulation through the two canonical transcription regulators AmtR and GlnR in response to nitrogen availability.
• The data can be used to characterize global regulatory networks in mycobacteria.

## 1 Data

Both raw and processed microarray data have been deposited in ArrayExpress with the Accession Number E-MTAB-4857 (https://www.ebi.ac.uk/arrayexpress/experiments/E-MTAB-4857/). The dataset summarize changes in the transcription profile of a *Mycobacterium smegmatis amtR* mutant compared to *M. smegmatis* wild-type. Raw data files are the result of four independent biological replicates including two dye swaps and the columns IA and IB in the raw data files correspond to Cy3 and Cy5 values, respectively. The processed data file contains the combined differential gene expression data including statistical analysis.

## 2 Experimental design, materials and methods

### 2.1 Construction of *amtR* deletion mutant

For a detailed description for the *amtR* deletion mutant construction, see Petridis et al. [1].

### 2.2 Microarray analysis and quantitative real-time PCR

Total RNA from *M. smegmatis* wild-type and *M. smegmatis* JR258 ∆*amtR* was extracted as previously described [2]. The quality of the RNA (RIN>9) was confirmed with the Bioanalyzer 2100 and the concentration was determined using a NanoDrop ND-100 spectrophotometer. RT-PCR was performed using SuperScript III (Invitrogen) according to the manufacturers instructions for cDNA synthesis and Phusion High-Fidelity PCR Kit (New England Biolabs) for PCR. Microarray analysis was performed using arrays provided by the Pathogen Functional Genomics Research Center (PFGRC) funded by the National Institute of Allergy and Infectious Diseases using protocols SOP# M007 and M008 from The Institute of Genomic Research (TIGR) [3]. The arrays were scanned using a Genepix 4000 A scanner and images were quantified using Spotfinder (TIGR) [3]. Total normalization and LOWESS normalization of the data were performed with the MIDAS software (TIGR) [3]. All data have been deposited in ArrayExpress with the Accession Number E-MTAB-4857.
